# Evaluation of hair and nail cortisol concentrations and associations with behavioral, physical, and environmental indicators of chronic stress in cats

**DOI:** 10.1111/jvim.16283

**Published:** 2021-10-27

**Authors:** Elena T. Contreras, Raphael Vanderstichel, Claire Hovenga, Michael R. Lappin

**Affiliations:** ^1^ Department of Veterinary Clinical Sciences Long Island University College of Veterinary Medicine Brookville New York USA; ^2^ Humane Society of Pikes Peak Region Colorado Springs Colorado USA; ^3^ Center for Companion Animal Studies Colorado State University Fort Collins Colorado USA

**Keywords:** animal welfare, cat, claws, enzyme immunoassay, fur, noninvasive measures, poor coat condition

## Abstract

**Background:**

Chronic stress is implicated in behavioral and health issues in cats, but methods for recognition, evaluation, and measurement of stress are lacking. Cortisol concentration is typically used as an indicator of stress.

**Objectives:**

To evaluate use of an enzyme immunoassay to quantitate hair and nail cortisol concentrations (HCC and NCC) in cats and evaluate associations between HCC and NCC and behavioral, physical, and environmental correlates of chronic stress in cats.

**Animals:**

Forty‐eight adult, owned or community cats.

**Methods:**

Cross‐sectional study. Nail clippings and hair were collected from cats. Medical history and cat daily lifestyle questionnaires were completed by owners or caretakers. A commercial laboratory performed cortisol extraction and quantification using a validated enzyme immunoassay kit. Correlational and regression analyses were used to evaluate associations between HCC and NCC and behavioral, environmental, and medical factors.

**Results:**

Hair and nail cortisol concentrations were significantly associated (*r*_s_ = 0.70; *P <* .001), but HCCs varied widely within and among cats. Cats with litterbox issues had significantly increased HCC (*P* = .02) and NCC (*P* = .001) as compared to cats without litterbox issues. Cats with groomed coats had lower HCCs (*P* = .02) as compared to cats without groomed coats, whereas cats with dander and mats had higher NCCs (*P* = .01) as compared to cats without dander and mats.

**Conclusions and Clinical Importance:**

The quantification of NCCs might improve identification and evaluation of chronic stress in cats. The variability of HCCs in individual cats warrants caution using this measurement in chronic stress studies.

**Abbreviations**
CCC: concordance correlation coefficient
CV: coefficients of variation
EIA: enzyme immunoassay
HCCs: hair cortisol concentrations
NCCs: nail cortisol concentrations
RIA: radio immunoassay
UC BIEL: University of Colorado Behavioral Immunology and Endocrinology Laboratory
VIF: variance inflation factor

## 1 INTRODUCTION

Stress contributes to illnesses in cats including upper respiratory infection, elimination disorders, dysrexia, grooming alterations, and gastrointestinal disturbances, often leading to additional medical sequelae.^1^, ^2^, ^3^, ^4^ Chronic stress also can initiate and potentiate common behavioral problems in cats, such as house soiling or aggression.^3^, ^5^, ^6^, ^7^ Cats frequently are surrendered to shelters because of these issues and may experience chronic stress when housed long‐term in a shelter.^8^, ^9^, ^10^, ^11^ Owners, however, might not notice signs of stress in their cats, and they might not attribute certain behaviors or illnesses to chronic stress, and thus it can go unrecognized.^12^, ^13^

Methods for recognition, evaluation, and physiological measurement of chronic stress in the cat are lacking. Quantitative stress measures in the cat historically have been based on serum cortisol concentration and more recently salivary, urinary, and fecal cortisol concentrations.^14^, ^15^, ^16^, ^17^, ^18^ None of these cortisol measurements as single results is indicative of chronic stress, however, because each gauges either instantaneous cortisol concentrations that can vary within minutes (as in serum and saliva), or concentrations that reflect the previous several to 24 hours (as in urine or feces).^16^, ^19^, ^20^ Furthermore, some of the procedures used to obtain the samples can induce stress in cats.^14^, ^21^

The slow growth rate and accumulation of cortisol within hair and nails allow for a single measurement of cortisol that reflects bodily concentrations of the hormone over an extended period of time.^22^, ^23^, ^24^, ^25^ Hair cortisol concentration (HCC) increasingly is being evaluated as an indicator of long‐term chronic stress in other species, and nail cortisol concentration (NCC) also is being evaluated.^22^, ^26^, ^27^, ^28^, ^29^ Enzyme immunoassays (EIAs) and radioimmunoassays (RIAs) have been used to measure HCCs in dogs,^16^, ^30^, ^31^ and RIA has been used to measure HCCs in cats.^32^, ^33^, ^34^ Coat color^31^ and body location^35^ might influence HCCs; these factors have not been assessed in cats. No published data are available regarding EIA assessment of HCC in cats. Nail cortisol concentrations have been found to be associated with HCCs in dogs^24^, ^36^ and had less variability than HCC.^24^ Nail cortisol concentrations have not been evaluated in cats.

Noninvasive objective measurements of chronic stress are needed in feline medicine. Hair and nail cortisol assays combined with observations, such as coat condition,^11^ might assist with evaluation of chronic stress in cats. Such measurements would more effectively guide prevention, intervention, and management strategies. Our aims were to evaluate use of EIA for the quantitation of HCC and NCC in cats and to determine if either could be used to evaluate chronic stress in cats. The primary hypotheses were that HCCs from different body locations would be similar and that HCCs would be positively associated with NCCs. The secondary hypotheses were that behavioral and physical factors potentially indicative of chronic stress in cats would be associated with HCC or NCC, and NCC would be more consistent than HCC.^24^

## 2 MATERIALS AND METHODS

### 2.1 Cats and samples

**FIGURE 1:**
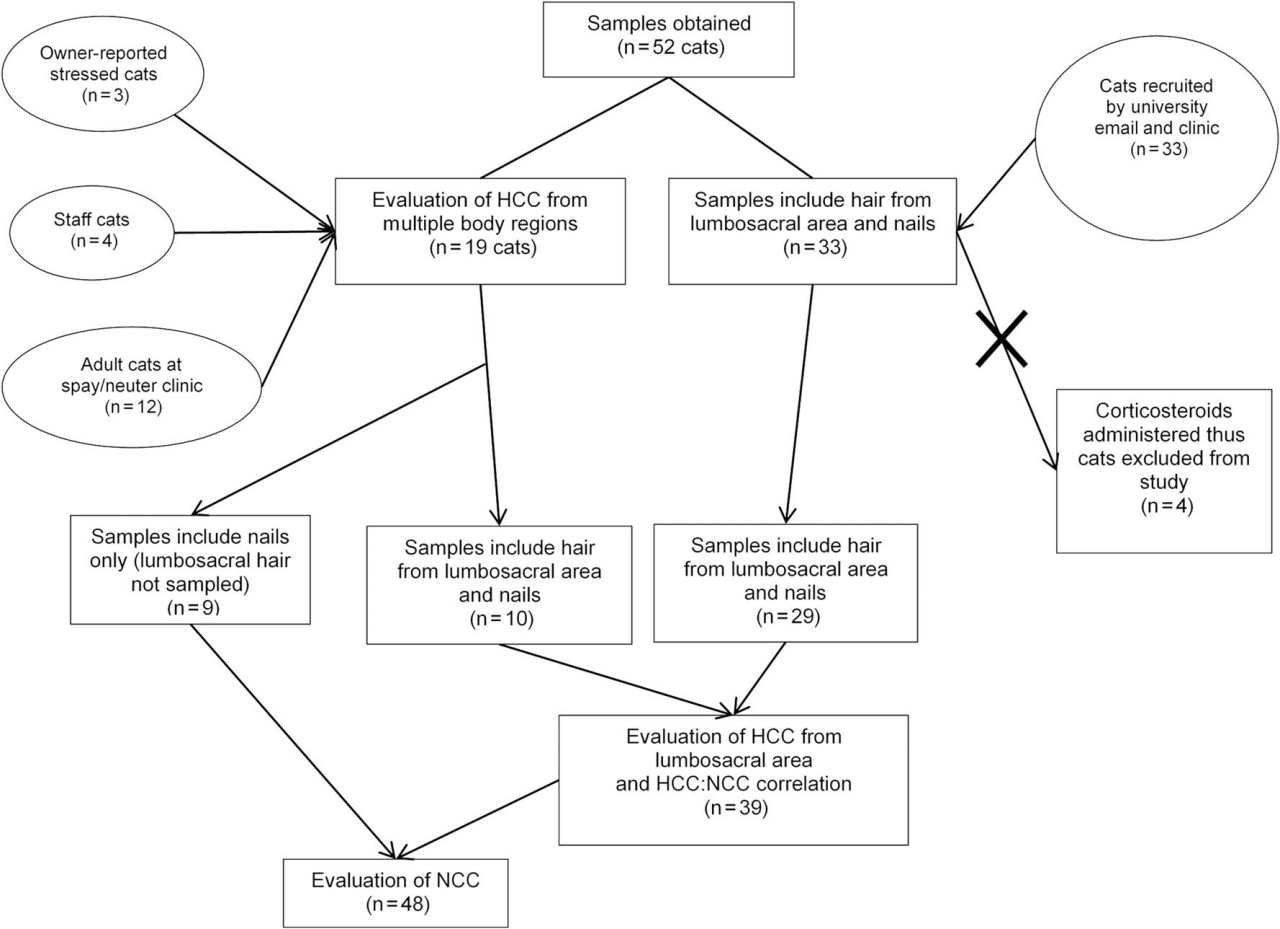
Flow diagram outlining cats recruited for study (ovals) and hair and nail sample inclusion and exclusion in different parts of the study and analyses (rectangles)

The subjects were either owned or community cats. Participants were recruited in 3 stages. At the request of the commercial laboratory, the first 3 cats were chosen based on owner report of being likely chronically stressed; this request was to potentially extract high concentrations of cortisol from the 3 samples to perform assay calibrations. The next 16 cats were healthy adult cats from teaching hospital staff and a spay/neuter clinic (Figure 1). Inclusion criteria were the following: generally healthy, adult, and no known glucocorticoid administration or contact within the past month, and if a cat had a nail trim performed within the past 2 weeks, it was excluded from participation. To enroll cats with a variety of owner‐reported health and behavioral conditions, participation next was sought from adult cats (n = 33) that had any of the following: geriatric age, unkempt appearance, excessive grooming behaviors, litterbox issues, recurrent upper respiratory clinical signs, chronic illness, owner‐reported stress, or some combination of these. If a cat had a nail trim performed within the past 2 weeks, it was excluded from participation. These remaining 33 cats were recruited via email request to veterinary students and university staff and from cats visiting a low‐cost feline clinic for grooming under sedation or euthanasia. Samples were collected between March 2018 and January 2019, and each cat's hair and nail samples were collected on the same day. Low‐stress handling techniques were used during sample collection from awake cats.^37^

Hairs in patches approximately 2 × 2 cm were clipped from each cat. To compare HCCs from different body locations, the first 19 cats had hair samples clipped from different body locations (dorsal neck, ventral neck, lumbosacral area proximal to the tail base, abdomen) based on simplicity of retrieval and previous HCC studies in cats,^32^ dogs,^31^ and other mammals.^22^, ^38^ After the first 19 cats were sampled, all of the remaining cats' hair samples were collected from only the lumbosacral area, selected based on previous studies and ease of sampling from that location.^31^, ^32^, ^39^ Hair samples were stored individually in separate aluminum foil packets at room temperature until ready for transfer to the commercial laboratory (University of Colorado Behavioral Immunology and Endocrinology Laboratory [UC BIEL], Aurora, Colorado) for processing.

Nails were clipped just distal to the quick from all front and rear paw digits or only the rear paw digits on outdoor community cats or front‐declawed owned cats. All samples were stored at room temperature in separate plastic vials until ready for transfer to UC BIEL for processing.

### 2.2 Cortisol extraction and assay

Cortisol extraction and laboratory validation of the assay were performed by UC BIEL using previously published methods.^22^, ^40^, ^41^, ^42^ Between 20 and 40 mg from each sample was used for analysis. Each sample was washed with 100% isopropanol, dried, ground, and extracted.^40^, ^41^, ^42^ A commercial high sensitivity EIA kit (Salimetrics, LLC) was used; the lower limit of sensitivity was 0.007 μg/dL. Cortisol concentrations were determined according to the manufacturer's protocol and were presented as picograms of cortisol/milligram (pg/mg) of hair or nail. To perform laboratory assay validation calibrations, samples from 3 cats were used. Pooled samples were processed to create low and high internal assay controls for determination of intra‐assay and interassay coefficients of variation (CV). The mean CVs were 10.6% and 9.3% for the low and high controls, respectively, and intra‐assay CV was 1.5%. Assay cortisol extraction recovery was evaluated by spiking 3 hair samples and 1 nail sample. The mean cortisol extraction recoveries were 97.1% for the hair and 89.2% for the nails. For comparison to the expected values of the assay standard curve, hair samples were serially diluted from 1 : 1 to 1 : 32, and nail samples were serially diluted from 1 : 1 to 1 : 8. Linear regressions showed strong positive correlations between expected and observed cortisol concentrations for hair (mean *R* ^2^ = 0.989) and nails (*R* ^2^ = 0.997).

### 2.3 Owner questionnaires

The owners or caretakers of the cats completed a medical history questionnaire and a daily lifestyle questionnaire based on previous studies.^12^, ^43^, ^44^ Questionnaires were not completed if the cat was being euthanized or was an unfamiliar community cat. Information gathered included signalment, owner‐reported medications the cat had received within the past month, current or chronic illness, observed clinical signs associated with respiratory or gastrointestinal disease (eg, sneezing, epiphora, diarrhea), retroviral status, neuter status, last nail trim, and house soiling. Other owner‐reported information included source of acquisition of cat, indoor vs outdoor housing, availability of resting and perching areas, number of adult humans and children in the household, number of other pets in the household, ratio of litterboxes to cats, occurrence of any recent household changes, in‐home pheromone use, owner‐reported cat personality characteristics, cat's coat condition and appearance, frequency of cat's activities (eg, playing, using scratching post, grooming, allogrooming, overgrooming, hissing, fighting with other cats, hiding, greeting owner, consuming inappropriate objects), and owner‐assessed stress level on a continuous gradient scale from not stressed at all to extremely stressed. The following factors were reclassified into categorical variables with 3 levels indicating frequency of behavior: uses scratching posts, rubs affectionately on owner, fights with or hides from other cats in home, and plays. Last nail trim also was categorized into 3 categories (between 2 and 4 weeks; >1 month; never). The following were reclassified into the dichotomous category of yes or no: children in household, pheromone use, litterbox issues (defecating or urinating outside of box or both), water spray punishment, pica, hisses or bites, greets when entering home, and chronic illness (including kidney disease, emaciated community cat euthanized, chronic rhinitis, chronic gingivostomatitis, severe periodontal disease, untreated asthma, thyroid disease, and obesity).

### 2.4 Statistical analyses

Statistical analyses were performed using Stata v14.2 (StataCorp LP). Significance was defined as *P* < .05. For the first 19 cats, the concordance correlation coefficient^45^ (CCC) and Bland‐Altman^46^, ^47^ plot were used to evaluate agreement between cortisol concentrations between hair collected from the dorsal neck compared to the lumbosacral area (n = 10) and the dorsal neck compared to the abdomen (n = 6) in paired samples; descriptive statistics were used to evaluate HCC from the ventral neck (n = 5). For the 3 cats that had >1 cortisol concentration assigned per body location for the purpose of assay calibration, the lowest of the results was used, and descriptive statistics also were used.

Spearman's rank correlation was used to evaluate the association between HCC (lumbosacral area) and NCC. Correlational matrices and univariable regression models were used to assess the association of cat characteristics, environmental conditions, and medical factors with HCC and NCC. Factors associated in the univariable analyses with a *P* ≤ .15 were used to build a multivariable regression model using manual bidirectional elimination. Clinically relevant eliminated variables were re‐evaluated for confounding effects within the final model, and collinearity was further evaluated using variance inflation factors (VIFs). The model and residuals were evaluated for normality, homoscedasticity, and linearity. Power transformations based on Box‐Cox analysis were applied to correct for violations of assumptions.

## 3 RESULTS

### 3.1 Cats and general characteristics

Samples were collected from 52 cats (Figure 1). Four cats were excluded from analyses because of topical corticosteroid contamination in the home (n = 3) and PO prednisolone administration (n = 1). Of the 48 cats that were used for the study, 5 were intact males (10.4%), 7 intact females (14.6%), 23 neutered males (47.9%), and 13 spayed females (27.1%). The median age of cats was 11.8 years old (range, 1.5‐20.0 years). Ten of the 48 cats (20.8%) were feral or community cats.

### 3.2 HCCs and body locations (n = 19)

**FIGURE 2:**
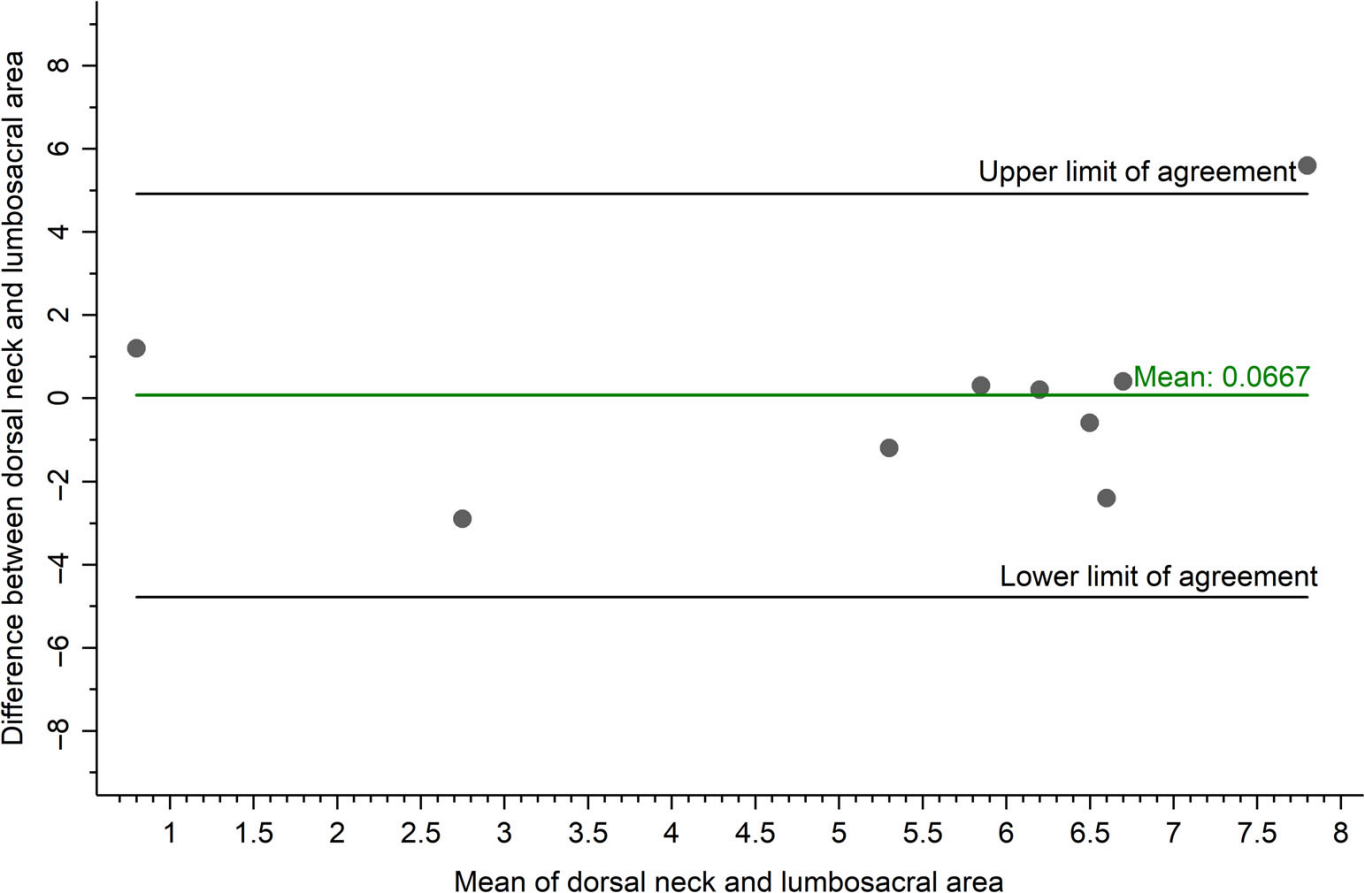
Bland‐Altman plot evaluating agreement between hair cortisol concentration (pg/mg) from the dorsal neck and lumbosacral area of the cat (n = 9; after removing outlying data from 1 cat)

**FIGURE 3:**
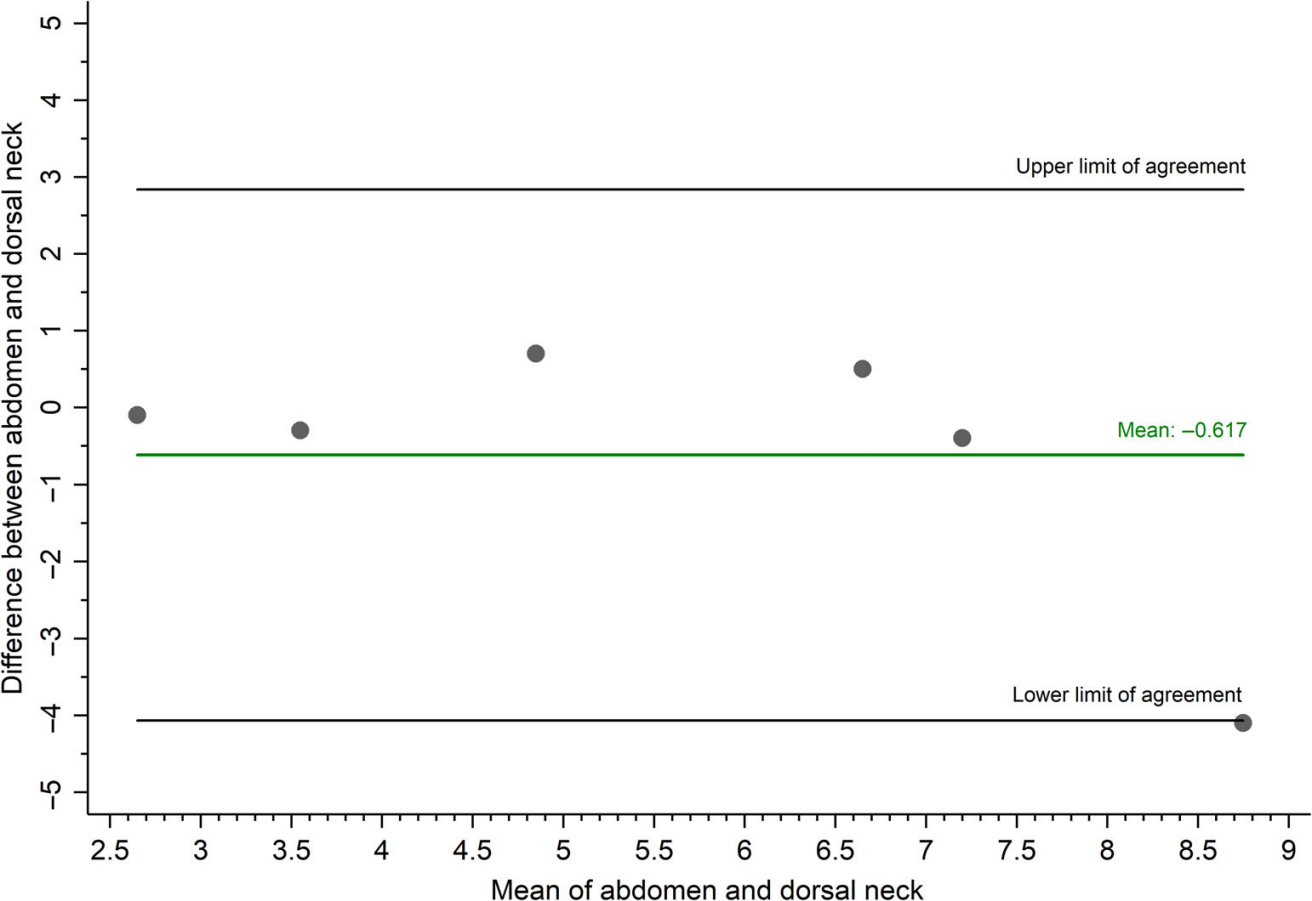
Bland‐Altman plot evaluating agreement between hair cortisol concentration (pg/mg) from the abdomen and dorsal neck (n = 6)

Samples were obtained from at least 1 body location in the first 19 cats (Table 1). The CCC comparing HCC from the dorsal neck to HCC from the lumbosacral area was 0.39 (95% confidence interval [CI],  0.28‐0.50; n = 10), indicating poor agreement between measures.^45^, ^46^, ^48^ After outlying data from 1 cat was removed from the analyses, the CCC was 0.53 (95% CI,  0.01‐1.04; n = 9), indicating improved agreement (Figure 2). The CCC comparing HCC from the dorsal neck to HCC from the abdomen was 0.72 (95% CI,  0.36‐1.09; n = 6), indicating moderate agreement (Figure 3).^45^, ^46^, ^48^

**TABLE 1:** Summary statistics for hair cortisol concentrations (pg/mg) by body location from which hair samples were obtained from the first 19 cats

Body location	Number of cats	Median (range)
Abdomen	6	5.5 (2.7‐10.8)
Dorsal neck	17	6.2 (1.3‐52.5)
Ventral neck	5	17.7 (3.0‐224.1)
Lumbosacral	10	6.3 (0.2–251.5)

### 3.3 Association between HCC from lumbosacral area and NCC (n = 39)

A significant positive association was found between HCC and NCC (*r*_s_ = 0.70; *P <* .001).

### 3.4 HCC from lumbosacral area and associations with behavioral, physical, and environmental factors (n = 39)

Median HCC from the lumbosacral area (n = 39) was 4.2 pg/mg (range, 0.2‐251.5 pg/mg). Median hair sample length was 30 mm (range, 17‐60 mm). Box‐Cox analysis indicated that a natural log transformation (with an offset of 1) on HCC would improve model fit. Univariable regression modeling identified multiple variables associated (*P* ≤ .15) with changes in HCC (Table 2). These variables were further evaluated using multivariable regression modeling. The final log‐transformed multivariable regression model retained 2 factors (litterbox issues; groomed and soft hair coat) that predicted HCC (*P* = .003; *R* ^2^ = 0.366; adjusted *R* ^2^ = 0.315). The VIFs for both variables in the final model were 1.01. In the final model, cats with litterbox issues had higher HCC (*P* = .02) as compared to cats without litterbox issues. Conversely, cats with groomed and soft hair coat were more likely (*P* = .02) to have decreased HCC as compared to cats without groomed and soft hair coat (Table 3). The HCC was not significantly associated with coat color (*P* = .46), sample color (*P* = .18), hair sample length (*P* = .78), sex (*P* = .67), neuter status (*P* = .62), indoor vs outdoor status (*P* = .38), ratio of litterboxes to cats (*P* = .77), or number of cats in the household (*P* = .21). After accounting for other contributing factors in the model, sample color, declaw status, chronic illness, number of dogs, owner‐reported stress levels, and other behavioral characteristics were not associated with HCC.

**TABLE 2:** Summary of univariable analysis with log transformations of hair cortisol concentrations (pg/mg) for 39 cats (Figure 1)

Variable	Level	Number	*P* value
Categorical variables			
Chronic illness	No	16	.08
	Yes	23	
Dander and mats	No	17	.03^b^
	Yes	18	
Declaw status	No	34	.02^b^
	Yes	5	
Feral or community cat	No	30	.14
	Yes	9	
Friendly, engaging demeanor	No	9	.01^b^
	Yes	21	
High perches available	No	10	.05
	Yes	22	
Home is quiet	No	6	.08
	Yes	24	
**Litterbox issues** ^a^	No	18	.02^b^
	Yes	10	
Plays	Never	4	.09
	Sometimes	5	
	Always	21	
**Soft and groomed hair coat** ^a^	No	10	.06
	Yes	28	
Continuous variables			
Number of dogs	0‐4	30	.02^b^
Stress axis (owner assessment)	0‐80	30	.03^b^

*Note*: Coefficients and confidence intervals are not included due to log transformations.

^a^ Included in multivariable final model.

^b^ Statistically significant at *P* < .05.

**TABLE 3:** Back‐transformed adjusted predictions of hair cortisol concentration (HCC) from the two significant variables, groomed and soft hair coat and litterbox issues in the final regression model for HCC

Groomed and soft hair coat	Litterbox issues	HCC magnitude prediction (pg/mg)
No	Yes	41.41
No	No	15.06
Yes	Yes	6.40
Yes	No	2.33

### 3.5 NCC and associations with behavioral, physical, and environmental factors (n = 48)

Median NCC (n = 48) was 0.6 pg/mg (range, 0‐10.3 pg/mg). Box‐Cox analysis indicated that an inverse transformation (with an offset of 1) on NCC would improve model fit. Univariable regression modeling identified multiple variables associated (*P* ≤ .15) with changes in NCC (Table 4). These variables were further evaluated using multivariable regression modeling. The final multivariable regression model retained 2 factors (litterbox issues, dander and mats) that predicted NCC (*P* < .001; *R* ^2^ = 0.379; adjusted *R* ^2^ = 0.341). The VIFs for both variables in the final model were 1.01. In the final model, cats with litterbox issues (*P* = .001), and cats with dander and matted hair coat (*P* = .01) had higher NCCs when compared to cats without litterbox issues and without dander and matted hair coat, respectively (Table 5). The NCC was not significantly associated with sex (*P* = .57), indoor vs outdoor status (*P* = .9), ratio of litterboxes to cats (*P* = .72), number of cats in the household (*P* = .85), or sneezing (*P* = .38). After accounting for other contributing factors in the model, age, intact status, declaw status, last nail trim, chronic illness, number of dogs, owner‐reported stress levels, and other behavioral characteristics were not associated with NCC.

**TABLE 4:** Summary of univariable analysis with transformed ([NCC + 1]^−1^) nail cortisol concentrations (pg/mg) for 48 cats (Figure 1)

Variable	Level	Number	*P* value
Categorical variables			
Chronic illness	No	23	.001^b^
	Yes	25	
**Dander and mats** ^a^	No	25	.001^b^
	Yes	19	
Declaw status	No	42	.01^b^
	Yes	6	
Fights with or hisses at other cats	Never	19	.06
	Sometimes	11	
	Always	5	
Feline immunodeficiency virus (FIV) positive	No	25	.12
	Yes	7	
Friendly, engaging demeanor	No	14	.01^b^
	Yes	25	
Greets owner	No	5	.1
	Yes	32	
Home is quiet	No	10	.04^b^
	Yes	28	
Intact reproductive status	No	36	.01^b^
	Yes	12	
**Litterbox issues** ^a^	No	24	.01^b^
	Yes	12	
Nails last trimmed	Never	12	
	Greater than 1 mo	22	.03^b^
	Between 2 and 4 wk	7	
Soft and groomed hair coat	No	11	.01^b^
	Yes	36	
Continuous variables			
Age (y)	1.5‐20.0	48	.001^b^
Number of dogs	0‐4	37	.06
Stress axis (owner assessment)	0‐80	38	.12

*Note*: Coefficients and confidence intervals are not included due to box cox transformations.

Abbreviation: NCC, nail cortisol concentration.

^a^ Included in multivariable final model.

^b^ Statistically significant at *P* < .05.

**TABLE 5:** Back‐transformed adjusted predictions of nail cortisol concentration (NCC) from the two significant variables, dander and matted hair coat and litterbox issues in the final regression model for NCC

Dander and matted hair coat	Litterbox issues	NCC magnitude prediction (pg/mg)
Yes	Yes	1.79
Yes	No	0.76
No	Yes	0.87
No	No	0.34

## 4 DISCUSSION

Hair and nail cortisol concentrations in cats were quantified using EIA in our study. The HCC and NCC were correlated, but HCC varied widely among and within body locations. The variable, dander and matted hair coat, was significantly associated with increased NCC, and the variable, groomed and soft hair coat, was significantly associated with decreased HCC. Both HCC and NCC also were increased in cats with litterbox issues as compared to cats without litterbox issues. Our study is the first to evaluate NCC in cats, and provides a preliminary evaluation of using noninvasive objective tools to evaluate long‐term cortisol concentrations as potential indicators of chronic stress in cats. Because of methodologic limitations and small sample sizes, these preliminary findings should be used as a starting point for further investigations.

It is important that veterinarians recognize signs of chronic stress in cats. One study noted that veterinary professionals' awareness of certain behaviors in cats was not significantly different from owners' awareness.^49^ If the nail cortisol assay becomes commercially available with reference intervals for cats, measurement of NCC could provide a valuable, mostly noninvasive, objective diagnostic tool to use during regular veterinary appointments or to gather additional information regarding housing in long‐term facilities such as shelters, catteries, or research facilities.

The amount of cortisol released into the hair or nail depends partly on the growth rate of the hair or nail.^16^, ^17^, ^25^ Hair growth rate in cats varies by season and hair type (primary or guard hair versus secondary or undercoat), but the average daily growth rate is approximately 0.33 mm/d for primary hairs and 0.25 mm/d for secondary hairs.^50^, ^51^ Because the median length of hair sampled in our study was 30 mm, the HCC reported might represent cortisol accumulation over approximately 3 months, but such extrapolations regarding the time period over which cortisol is deposited and accumulates in the hair shaft represent speculations and warrant further exploration. Nail growth rates in cats have only been extrapolated from rates in dogs in the literature.^52^, ^53^ Unpublished data by one of us (E.T. Contreras) found nail growth rate in the cat to be approximately 2.4 mm for front nails and 1.7 mm for rear nails per 21 days, at which time nails were full and pointed. The portion of the nail available for trimming, however, is not the most proximal portion of the nail in contact with the nail matrix, and thus the cortisol concentration obtained from the trimmed portion might represent a prior time period. Although we did not measure the length of the nails trimmed, cats that had nails trimmed within the prior 2 weeks were excluded from participation, and all cats therefore had full and pointed nails upon trimming for the study. The NCC reported therefore might represent cortisol accumulation for an approximately 21‐day period at least 1 month before trimming. The physiology of the deposition of cortisol into the nail matrix and nail structure, however, has not been explored in cats and warrants future investigation.

Our hypothesis that HCCs from different body locations would be similar was not supported, and HCC occasionally varied widely within an individual cat at a single point in time, however sample sizes for this part of the study were very small. Similar to our study, several other recent studies have shown that hair in different regions of the body in various species has variable concentrations of cortisol.^35^, ^38^, ^54^, ^55^ The HCC of the Canada lynx was found to vary among foot, leg, location on foot or leg, and by individual,^55^ and significantly different HCCs in body regions also have been found in grizzly bears, with neck having the highest cortisol concentration and rump the lowest.^35^ In contrast, 1 study that evaluated HCC in dogs did not report variation between right and left shoulder HCCs.^30^ Variation in HCCs also might be partly attributable to different concentrations in guard vs undercoat hairs.^35^, ^56^, ^57^ Variation also might be a consequence of >1 source of cortisol from both the skin's localized stress response as well as from systemic circulating cortisol.^32^, ^58^, ^59^, ^60^

Neither hair segment length nor color were responsible for the variation in HCC in our study, because those variables were not significant in analyses, but sample sizes were too small within each color category to draw conclusions. Conflicting findings have been reported regarding these factors in other studies.^31^, ^35^, ^54^ It was found that dogs had higher HCC in the distal segment of hair as compared to the proximal segment,^31^ whereas chimpanzees had significantly decreasing HCC toward the distal end of the hair shaft.^54^ Conflicting results also have been reported regarding the relationship between hair color and HCC.^31^, ^35^

The final HCC model in our study was driven, in large part, by a small number of cats with extremely high HCC. Hair cortisol concentrations in cats have been evaluated by RIA in 4 previous studies,^32^, ^33^, ^34^, ^39^ 1 of which reported a wide range in HCC of 0.02 to 76.27 pg/mg.^34^ Although our study used EIA and not RIA, and different assays may produce different results,^61^ HCCs determined by either method have sometimes disclosed wide ranges in HCCs in other species^31^, ^56^, ^57^, ^61^, ^62^ and for samples obtained at different times of the year,^35^, ^38^, ^54^, ^55^ whereas others have not found such inconsistencies.^30^ The reliability of any singular measurement of HCC in the cat is questionable considering the matrix of potentially different cortisol concentrations depending on source, variable hair growth rates, types, amounts, densities, and physiology.

The amount of cortisol extracted from nails was lower than that extracted from hair in our study. Lower NCCs as compared to HCCs also have been reported in dogs, and it was suggested that there might be a slower rate of diffusion from circulation into the nail as compared to the hair follicle.^24^ Cat nails are unique in morphology, function, and structure with a complex architecture of multiple layers, vasculature, growth rates, and shedding mechanisms.^23^, ^63^, ^64^ The diffusion of hormones to the nails might not occur at the same rate and volume as in the hair.^65^ As the amount of cortisol released into and accumulated in nails depends, in part, on nail growth rate, the rapid growth rate might be partly responsible for lower cortisol concentrations in nails vs hair in cats. Because the unique structure of the feline nail likely leads to difficulties in extracting large amounts of cortisol, improved methods of extraction should be explored.

Cats' front limbs and paws differ from the rear limbs and paws in function, musculature, and innervation.^66^, ^67^, ^68^ Cats' front nails therefore might differ from rear nails in blood supply and deposition of hormones as well as in growth rate and cortisol accumulation. Unpublished preliminary data by one of us (E.T. Contreras) found that front nail growth rate in the cat might be higher than rear nail growth rate. Interestingly, however, our preliminary findings^69^ with a less robust model found that front‐declawed cats had higher cortisol concentrations than cats that were not declawed. The preliminary model, however, did not account for multiple variables, and front vs rear nail comparisons were not sufficiently evaluated. The more robust final model reported here accounted for such factors, and neither declaw status nor “nails used” (all vs rear only) was significantly associated with cortisol concentrations. However, because only rear NCCs were evaluated in 13 cats, caution in interpretation is warranted. Future studies should evaluate front vs rear NCCs in cats.

Our results supported the hypothesis that HCC and NCC were positively correlated, although NCCs did not have as much variation as HCCs. Similarly, in the 1 study that evaluated adult dog hair and nails, outlying values were found in the hair data, but in not the nail data.^24^

Findings also supported our secondary hypotheses that behavioral and physical factors would be associated with HCC and NCC. Litterbox issues and poor hair coat were associated both with HCC and NCC in the final models. Our findings are supported by other studies that suggested that house soiling and poor hair coat are related to cats' emotional and physical health.^11^, ^70^

The variable, groomed and soft hair coat, was associated with decreased HCC, whereas the variable, dander and matted hair coat, was associated with increased NCC. Chronically stressed cats often have unkempt appearances because they discontinue performing regular behaviors such as grooming; lack of grooming can indicate a medical or stress‐related issue or both.^11^, ^71^, ^72^ One shelter study found that poor hair coat was associated with lack of enrichment, limited access to resources, overcrowding, and longer shelter stays.^11^ A feral cat colony study found poor hair coat was associated with larger colony size and number of cats per feeding station, indicating heightened social tensions and less access to resources.^73^ High behavioral stress scores also have been found in cats with decreased grooming behaviors.^74^ Some cats cannot groom because of medical conditions including chronic pain, and these cats also might be experiencing chronic stress because of those conditions. Therefore, hair coat condition should be monitored as an indicator of overall welfare in cats.

Our study had several limitations. The small sample size could lead to type 2 errors, whereas using multiple variables to predict a best fit model with a small sample size could result in type 1 errors.^75^, ^76^ Another limitation was that it was not an experimental study evaluating presumably stressed as compared to nonstressed cats, but an observational study, lacking a dedicated control group, and the subjects had varied histories. If future studies further evaluate hair from different body areas, hair should be collected more systematically so that improved comparisons among body areas can be made. Although cat HCC has been evaluated in 4 other studies, understanding the timeframe over which cortisol accumulates in the hair in cats is still not validated, and no other study of cat NCC has been published to our knowledge.

Another limitation was that the medical, behavioral, and environmental data were obtained only through owner reporting, which could have resulted in bias and inaccurate reporting.^77^, ^78^ Many of the subjective owner‐assessments of cats in our study were not associated with cortisol concentrations. Results might suggest that some owners were unaware of their cats' affective states or stress levels. In a study of the perception of stress in cats, owners did not recognize some important indicators, and most considered their cats to have a very low stress level.^12^ Another study found that owners did not utilize many recommended environmental modifications and enrichment modalities and were unaware of the adverse consequences.^77^ It is also possible that owner reporting is inaccurate when describing environmental enrichment modalities. So that owners know when to seek further care for their cat, it is important that they understand the importance of the cat's environment, behaviors indicative of a cat with good versus poor quality of life, and recognition of signs of acute and chronic stress. Future studies should consider obtaining objective laboratory test results to determine health and chronic disease and a full behavioral evaluation including in‐home assessment by a trained behaviorist or veterinarian to determine the behavioral and mental health of the cat. Additional studies with larger sample sizes also should be conducted.

Chronic stress can severely and detrimentally impact the health, immune system, and welfare of a cat,^2^, ^79^, ^80^ yet many cats in a variety of settings likely experience variable degrees of chronic stress, often unrecognized and certainly unmeasured.^11^, ^81^ The quantification of NCC in cats combined with assessment of physical, behavioral, and environmental factors might improve identification and evaluation of chronic stress in cats. Although some cats experience acute distress related to nail trims, this temporary state should not interfere with measurement of long‐term NCC. In addition, simple observational measures such as unkempt appearance or litterbox issues also can be monitored to intervene accordingly. Having objective, noninvasive, and reliable indicators of chronic stress would substantially improve prevention, intervention, and monitoring strategies, ultimately substantially improving the welfare of cats. The variability of HCC in individual cats warrants caution be used when evaluating this measurement in studies of chronic stress in cats.

## CONFLICT OF INTEREST DECLARATION

Authors declare no conflict of interest.

## OFF‐LABEL ANTIMICROBIAL USE DECLARATION

Authors declare no off‐label use of antimicrobials.

## INSTITUTIONAL ANIMAL CARE AND USE COMMITTEE (IACUC) OR OTHER APPROVAL DECLARATION

The Colorado State University College of Veterinary Medicine and Biomedical Sciences Clinical Review Board approved this protocol.

## HUMAN ETHICS APPROVAL DECLARATION

Authors declare human ethics approval was not needed for this study.
